# Structural Insights in Mammalian Sialyltransferases and Fucosyltransferases: We Have Come a Long Way, but It Is Still a Long Way Down

**DOI:** 10.3390/molecules26175203

**Published:** 2021-08-27

**Authors:** Ravneet Kaur Grewal, Abdul Rajjak Shaikh, Suresh Gorle, Manjeet Kaur, Paula Alexendra Videira, Luigi Cavallo, Mohit Chawla

**Affiliations:** 1 STEMskills Research and Education Lab Private Limited, Faridabad 121002, Haryana, India; razzaqsk@gmail.com (A.R.S.); suresh4chem123@gmail.com (S.G.); 2 Kaust Catalysis Center, Physical Sciences and Engineering Division, King Abdullah University of Science and Technology (KAUST), Thuwal 23955-6900, Saudi Arabia; 3 Department of Biochemistry and Molecular Biology, University of Texas Medical Branch, Galveston, TX 77555, USA; 4 Biotechnology Engineering, University Institute of Engineering & Technology (UIET), Maharshi Dayanand University, Rohtak 124001, Haryana, India; manjeetkaurbiotech.uiet@mdurohtak.ac.in; 5 Associate Laboratory i4HB—Institute for Health and Bioeconomy, NOVA School of Science and Technology, NOVA University Lisbon, 2819-516 Caparica, Portugal; p.videira@fct.unl.pt; 6 UCIBIO—Applied Molecular Biosciences Unit, Department of Life Sciences, NOVA School of Science and Technology, NOVA University Lisbon, 2819-516 Caparica, Portugal; 7 CDG & Allies—Professionals and Patient Associations International Network (CDG & Allies—PPAIN), 2829-516 Caparica, Portugal

**Keywords:** sialyltransferase, fucosyltransferase, glyocosyltransferases in cancer, drug design

## Abstract

Mammalian cell surfaces are modified with complex arrays of glycans that play major roles in health and disease. Abnormal glycosylation is a hallmark of cancer; terminal sialic acid and fucose in particular have high levels in tumor cells, with positive implications for malignancy. Increased sialylation and fucosylation are due to the upregulation of a set of sialyltransferases (STs) and fucosyltransferases (FUTs), which are potential drug targets in cancer. In the past, several advances in glycostructural biology have been made with the determination of crystal structures of several important STs and FUTs in mammals. Additionally, how the independent evolution of STs and FUTs occurred with a limited set of global folds and the diverse modular ability of catalytic domains toward substrates has been elucidated. This review highlights advances in the understanding of the structural architecture, substrate binding interactions, and catalysis of STs and FUTs in mammals. While this general understanding is emerging, use of this information to design inhibitors of STs and FUTs will be helpful in providing further insights into their role in the manifestation of cancer and developing targeted therapeutics in cancer.

## 1. Introduction

The glycome, the complex glycan repertoire of the cell, is involved in a myriad of cellular events in health and disease [1,2,3,4,5]. Unlike the genome, transcriptome, and proteome, glycan biosynthesis is not template-driven but is determined by the location and coordinated activities of the glycan processing enzymes, glycosyltransferases (GTs) and glycoside hydrolases (GHs), and the availability of their substrates. Structural analysis of these two glycan-processing enzyme families highlighted that GHs exhibit a vast diversity of three-dimensional (3D) scaffolds, despite common features in their active sites, indicating an independent convergence during evolution [6]. As for GTs, this trend seems divergent, and three general folds, named GT-A, GT-B, and GT-C, have been reported for glycosyltransferases [7,8,9,10].

GTs are multi-substrate enzymes that transfer the donor’s sugar moiety, mostly an activated nucleotide, to the acceptor molecule, which is a glycan, a protein, or a lipid molecule. In eukaryotes, most GTs are type II transmembrane proteins with a short N-terminal domain, a transmembrane domain followed by a stem region, and a large C-terminal catalytic domain at the luminal side [8]. GTs are classified into 114 families in the Carbohydrate-Active enZYme (CAZy) database (available at http://www.cazy.org/) Accessed on 24 April 2021 [11]. This sequence-based classification intercalates structural and mechanistic characteristics within each GT family and highlights that monofunctional GTs have similarities in terms of amino acid sequences for the overall catalytic domain. However, many GT families are polyfunctional, comprising different kingdoms of life, e.g., the GT2 family, which contains more than 2.4 × 10^5^ sequences, originating from animal, plant, yeast, and bacterial species, and exhibits sequence heterogeneity except for a portion of the catalytic domain. It is noteworthy that the functional prediction of putative GTs (e.g., an open reading frame) can be challenging. This is because many closely related sequences reported in the CAZy database, especially those related to polyfunctional GT families, may perform different catalytic functions [12].

There are numerous challenges toward a structural understanding of GTs [13,14,15,16], which include the following: (a) the crystallization process is demanding since GTs are often multi-domain proteins and undergo considerable conformational changes; (b) it is difficult to produce a high yield in the recombinant form, especially for integral and membrane-bound GTs; (c) characterization is laborious as the identification of both donor and acceptor substrates is required; and (d) GTs undergo a series of post-translational modification events, such as N-glycosylation, disulfide bond formation, and proper folding assisted by chaperonins. Such modifications require eukaryotic expression systems, posing a big challenge to producing an enzyme in vitro. Recently, studies have attempted to solve this challenge using HEK293 cells, and significant progress has been achieved [14].

Sialyltransferases (STs) and fucosyltransferases (FUTs) are two distinct classes of GT in terms of structural fold architecture, substrate specificity, the nature of their interactions with both donor and acceptor substrates, and catalysis. Intriguingly, the dysregulation of both STs and FUTs results in the altered expression of sialylated and fucosylated epitopes, which are hallmarks of cancer cells [17,18,19,20,21,22,23,24,25]. To date, their chemical biology and potential for drug discovery in cancer have been relatively unexplored [15]. This is due, at least in part, to the relative lack of ST- and FUT-specific inhibitors for structural, mechanistic, and cellular studies. Furthermore, slow progress in inhibitors of STs and FUTs may be attributed to the complexity of GTs in terms of their polyspecific nature, overlapping specificities, and multi-substrate catalytic mechanism. Additional, relatively little 3Dstructural data is available, and there has been limited understanding of their catalysis until the past few years [15]. Thus, it is necessary to combine information on the structural fundamentals and advances in the understanding of the nature of enzyme–substrate binding interactions of mammalian STs and FUTs into a unified platform, which may be helpful for researchers working toward ST-and FUT-targeted drug discovery in cancer.

## 2. ST and FUT Families

### 2.1. Twenty STs Are Arranged into Four Families

**Figure 1:**
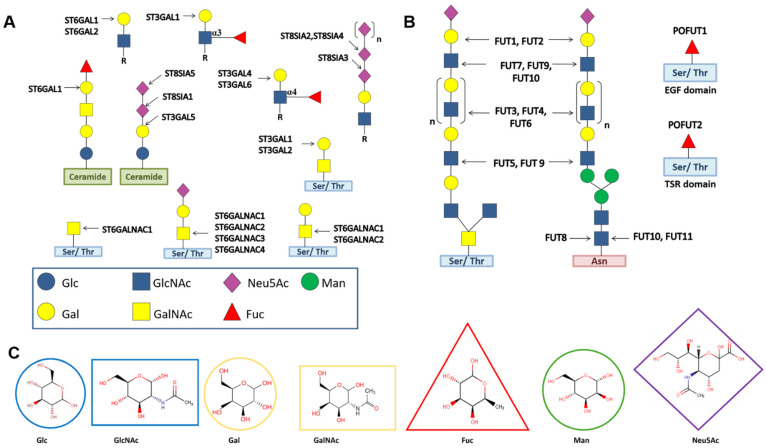
Sialylation and fucosylation sites of human STs and FUTs. (**A**) Sialylation site for STs including ST3GAL; ST6GAL; ST6GALNAC; and ST8SIA, which generates sialylated-tumor-associated carbohydrate antigens; (**B**) fucosylation site of human FUTs including α,2 FUT; α1,3/4 FUT; α1,6 FUT; and O-FUT. STs and FUTs transfer Neu5Ac and Fuc, respectively, to different monosaccharide residues of *O*-glycans;*N*-glycans; EGF or TSR domains of proteins, e.g., Notch and thrombospondin 1; and glycolipids, as indicated by the arrows. Abbreviations: Glc: D-Glucose; Gal: D-Galactose; GlcNAc: *N*-Acetyl-D-glucosamine; GalNAc: *N*-Acetyl-D-galactosamine); Fuc: L-Fucose; Man: D-Mannose; Neu5Ac: *N*-Acetyl-D- neuraminic acid (or sialic acid); Ser/Thr: serine/threonine; Asn: asparagine; EGF: epidermal-growth-factor-like repeat; and TSR: thrombospondin type 1 repeat. R represents *N-* or *O-*glycoproteins or glycolipids. (**C**) Structural representation of the monosaccharide residues present in glycans.

Depending on the regio-selectivity of the acceptor substrate in addition to *N*-Acetylneuraminic acid (Neu5Ac) as well as their linkages, STs are classified into four families: ST3GAL; ST6GAL; ST6GALNAC; and ST8SIA. Each family consists of several STs (Table 1). ST3GAL STs transfer Neu5Ac to the 3-OH of Gal residues of *N*-, *O*-linked glycans, and glycolipids. Members of the ST6GAL family catalyze the addition of Neu5Ac to the 6-OH of the Gal residue of *N*-glycans, while the STs of ST6GALNAC transfer Neu5Ac to the 6-OH of GalNAc residues of *O*-glycans and glycolipids. ST8SIA STs are the only STs that promote the transfer of Neu5Ac to the 8-OH of another Neu5Ac residue in *N-*, *O*-linked glycans, and glycolipids [26] (see Figure 1). STs in mammals have been grouped into GT29 in the CAZy database (Table 1). The cell sialylation status, which is determined by the extent and nature of sialic acid linkages, undergoes a transformation in cancer progression, which is correlated with the upregulation of sialyltransferases [17,19,21,22,23] (Table 1).

### 2.2. Sequence Analysis and Conserved Patterns in STs

The catalytic domain of all STs is characterized by four conserved peptide sequences, termed sialylmotifs: large (L), small (S), 3rd (III), and very small (VS) [27,28,29]. Human ST sequences have low sequence similarity but share 10 invariant residues—five in motif L, two in motif S and VS, and one in motif III [23,27]. Motif L is mainly engaged in binding donor substrates, while sialylmotifs S, III, and VS are involved in binding acceptor substrates or both substrates [23,27,30,31]. Both L and S contain an invariant cysteine residue and participate in the formation of an intramolecular disulfide linkage essential for the active conformation of the enzyme [32,33]. Mutational analyses with motifs III and VS highlighted the involvement of motif VS in catalysis. The sequence consensuses of motifs III and VS in human STs are ((H/y)-Y-(Y/W/F/h)–(D/E/q/g)) and H-x4-E (where lowercase/capital letters imply low/strong occurrence of the amino acid), respectively [28,34].

Multiple sequence alignment of STs in vertebrates revealed the presence of family motifs containing four to twenty amino acids specific to each ST family, thus implicating another level of amino acid conservation among STs [29,31,35]. Except for ST6GALNAC, all STs contain four common amino acid sequences located eight amino acids downstream of the 3′-end of sialylmotif L, termed motif “a”. The seven amino acids containing ST6GALNAC motif “a” are located four amino acids closer to sialylmotif L. Another family motif, “b”, lies 20 amino acids downstream from sialylmotif L and is present between sialylmotifs L and S. Interestingly, motif “b” is highly variable in length among ST families. Motif “c” is the another family motif with two amino acids overlap at the 3′-end of sialylmotif S a The family motif “d” is located downstream from sialylmotif III in ST6GALs while motif “e” is found downstream from sialylmotif VS in the ST8SIA and ST6GALNAC families.

### 2.3. Thirteen FUTs Are Organized into Four Families

Depending on their linkage specificities and acceptor substrates, 13 human FUTs are organized into four families—α1,2 FUT; α1,3/4 FUT; α1,6 FUT; and O-FUT (Figure 1). The α1,2 FUT family contains FUT1 and FUT2. α1,3/4 FUTs are classified into eight enzymes—FUT3, FUT4, FUT5, FUT6, FUT7, FUT9, FUT10, and FUT11. Interestingly, α1,6 FUT contains only FUT8, whereas the O-FUT family includes the O-fucosyltransferases POFUT1 and POFUT2 [25]. FUTs in mammals have been classified into four distinct families in the CAZy database (Table 1). FUT1 prefers type II (Galβ1,4GlcNAc) acceptors, while FUT2 has a specificity for type I (Galβ1,3GlcNAc) glycans [36,37]. FUT3 and FUT5 can utilize both type I and type II acceptors, and modification of an amino acid can alter their relative α1,3 or α1,4 specificities [38,39,40]. FUT3, FUT5, and FUT6 can transfer L-fucose (L-Fuc) to both non-sialylated and sialylated acceptors. FUT4 and FUT9 prefer non-sialylated type II acceptors, while FUT7 is specific for the sialylated type II acceptor [31,41,42]. FUT10 and FUT11 add L-Fuc onto the innermost GlcNAc residue of the chitobiose unit of biantennary *N-*glycans and are clearly distinguishable from the classical α1,3 FUTs (i.e., FUT3, FUT4, FUT5, FUT6, and FUT7), which utilize short and linear lactosamine-related acceptors [25,43].

The preferred site for fucosylation varies considerably among α1,2 and α-1,3/4 FUTs, which has a remarkable impact on the synthesis of terminally fucosylated epitopes, such as blood antigens (H1 and H2) and Lewis antigens (Le^x^, Le^y^, Le^a^, Le^b^, sLe^x^, and sLe^a^), thus contributing toward the complexity of fucose-containing Lewis epitopes in naturally occurring glycoconjugates [24,44]. Pronounced over-expression of Lewis epitopes has been reported in the manifestation of cancer [17,18,19,20,21,22,23,24,25] (Table 1). SialylLewis (sLe^x^ and sLe^a^) epitopes are selectin ligands that are overexpressed in cancers due to the upregulation of FUT4, FUT5, FUT6, and FUT7 [45]. sLe^x^/sLe^a^ contribute to cancer dissemination and metastasis [46] FUT8 promotes core fucosylation, which is ubiquitously present in N-glycans [47], whereas POFUT1 and POFUT2 catalyze the transfer of fucose to *O*-glycan and selectively fucosylate epidermal-growth-factor-like (EGF) repeats and thrombospondin type 1 repeats (TSRs) (Figure 1), respectively, present in the extracellular domain of several proteins such as Notch and thrombospondin 1 [48,49]. Aberrant core fucosylation is also reported to contribute to malignancies [50] (Table 1).

### 2.4. Sequence Analysis and Conserved Patterns in FUTs

Hydrophobic cluster analysis performed on FUTs from vertebrates, invertebrates, plants, and bacteria revealed conserved peptide sequences termed as FUT motifs—three in the catalytic region common to α1,2 and α1,6 FUTs [51,52]; two specific to α1,3/4 FUTs; one for α1,2 FUTs; and one unique to α1,6 FUTs. Interestingly, three motifs are shared by α1,2 FUTs, α1,6 FUTs, and O-FUTs that are common among the sequences of these families, including residues involved in binding the nucleotide of the sugar donor [52,53,54]. The α1,2, α1,6, O-FUTs, and α1,3/4 families of FUT are likely descended from a common progenitor. Interestingly, α1,3/4 FUTs have emerged as distant to the rest of the families [52,55]. Sequence alignment of α1,3FUTs revealed that 17 amino acids, FxL/VxFENS/TxxxxYxTEK, commonly referred to as the α1,3 motif, are highly conserved among species [41]. In the past few years, impressive progress has been made in the elucidation of structures of FUTs in eukaryotes, including mammals [56,57,58,59,60,61,62,63]. Boruah et al. performed structural alignments of FUTs and reported structural similarities far beyond the three previously identified motifs, α1,2 FUTs, α1,6 FUTs, and O-FUTs [52,53,54]. Furthermore, thirteen amino acids were identified that are the most conserved among these structures, few of which were noted in a more focused comparative analysis of the sugar donor binding region [51].

## 3. Cellular Localization of STs and FUTs

**Figure 2:**
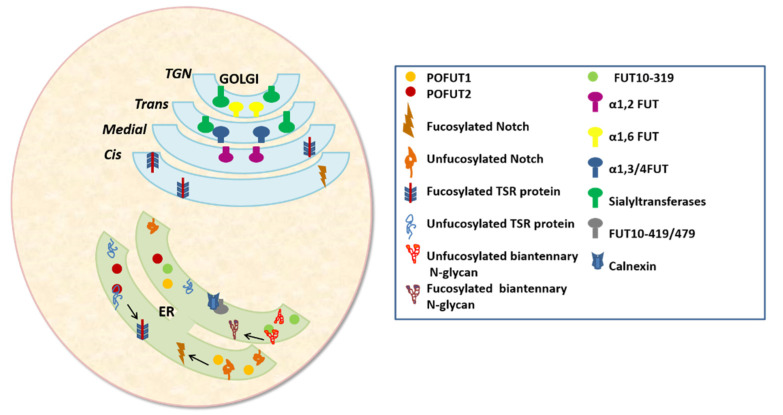
Cellular localization of STs and FUTs.α1,2 FUT,α1,3/4 FUT,α1,6- FUT, and all STs are type II membrane proteins anchored at the Golgi, except for O-FUTs and FUT10, which are retained at the ER.FUT10-319 is reported to be a soluble protein, while FUT10-419/479 appear to be membrane-bound in the ER.

As of 30 January 2021, the CAZy database annotated 242 GT sequences in the human genome organized into 47 GT families (http://www.cazy.org/) Accessed on 24 April 2021. Both STs and FUTs present a complex tissue-, cell-type-, and stage-specific expression pattern, and are expressed as both membrane-bound and soluble proteins [64]. Analogous to the other Golgi-resident GTs, all human STs and FUTs cloned to date typically share a type II architecture, containing an N-terminal transmembrane domain anchored at the Golgi membrane and a C-terminal catalytic region exposed to the Golgi lumen present in the late cisternae of the Golgi [40,65]. O-FUTs, on the other hand, are ER-localized soluble proteins which fucosylate Notch and TSR domains of proteins [66] (Figure 2). POFUT1 first fucosylates EGF domains in the ER and acts as a chaperone to aid protein secretion to the cell surface. Proteins with TSR domains are fucosylated by POFUT2, but whether this occurs in the ER requires further investigation [40]. Mollicone et al. [43] cloned three active isoforms of the human *FUT10* gene and investigated their subcellular distributions. The *FUT10*-319 isoform encodes a soluble protein expressed in human embryos. FUT10-419 and FUT10-479 are reported to be co-localized with calnexin (Figure 2), be retained in the ER, and be expressed in the human embryo and brain, respectively.

## 4. Global Fold Architecture

Emerging structural information on GTs in the past two decades has been consolidated into three catalytic domains of GTs, organized as GT-A, GT-B, and GT-C, while unresolved folds are characterized as orphans. GT-A and GT-B folds primarily consist of α-β-α sandwiches, analogous to the Rossmann fold. However, the third fold, GT-C, is the characteristic lipid-phosphate-dependent GT fold, containing multiple transmembrane α-helices [12,67,68,69]. Both GT-A and GT-B folds employ analogous approaches to interact with nucleotide sugar donor substrates, a similarity that is attributed to the constraints of the interacting loops that extend from the Rossmann fold. However, they vary considerably in terms of their interactions with acceptor substrates.

The architecture of GT-A is reminiscent of two tightly associated β/α/β Rossmann domains, the sizes of which vary, leading to the formation of a continuous central β-sheet to create the N-terminal donor and C-terminal acceptor binding regions [8]. Most GT-A enzymes display a DxD motif signature, where “x” represents any amino acid that coordinates divalent cations (typically Mn^2+^ or Mg^2+^) to the phosphate group of the nucleotide. It is noteworthy that the DxD motif is not a conserved feature of the GT-A fold, since there are examples of enzymes containing this fold that lack this motif [8].

The GT-B fold, on the other hand, consists of two separate β/α/β Rossman domains, i.e., an N- and a C-terminal domain separated by a large cleft where the active site is located and stabilized by two long C-terminal-helices. The GT-B fold lacks the DxD motif and generally does not require metal ions for catalysis. Donor and acceptor substrates bind to the C- and N-terminal regions of GT-B, respectively [8].

### 4.1. STs Display Variants of the GT-A Fold

**Figure 3:**
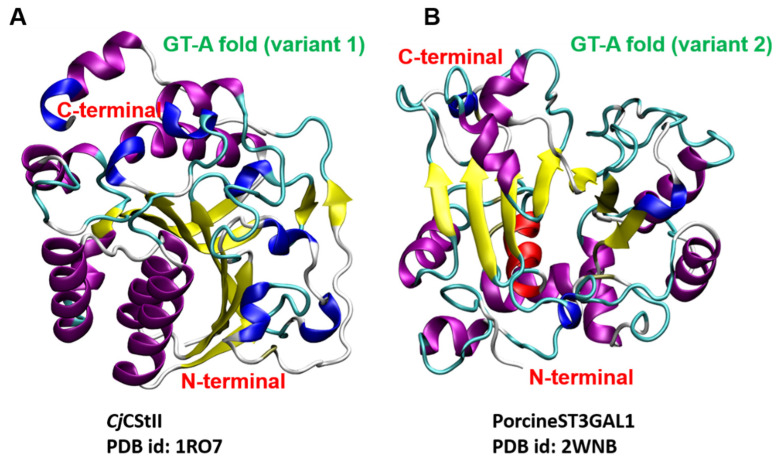
Representative glycosyltransferase global folds. (**A**) GT-A (variant 1) fold correspondingto *C. jejuni* CstII (PDB id: 1RO7); (**B**) GT-A (variant 2) fold corresponding to porcine ST3GAL1(PDB id: 2WNB).

**Figure 4:**
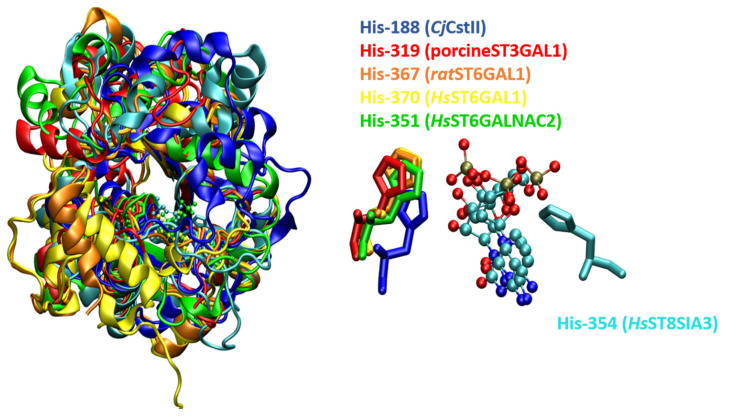
Structural superimposition of the backbone of sialyltransferases from *C. jejuni* CstII (blue, PDB id: 1RO7), porcine ST3GAL1 GAL1 (red, PDB id: 2WML), ratST6GAL1 (orange, PDB id:4MPS), *Hs*ST6GAL1 (yellow, PDB id: 4JS1), *Hs*ST6GALNAC2 (green, PDB id: 6APL), and *Hs*ST8SIA3 (cyan, PDB id: 5BO6), with the orientation and interaction pattern of histidine residues with the donor substrate.

Although mammalian STs belong to the GT29 family, intriguingly, they have been predicted to be similar to the CstII fold, a GT-A variant (i.e., variant 1) from *Campylobacter jejuni*, belonging to the GT42 CAZy family [27]. CstII is comprised of two closely associated domains. One domain has a mixed α/β fold with a central, parallel, seven-stranded, twisted β-sheet, flanked by helices on either side. The other domain is composed of a long coil and two helices forming a lid-like structure folded over the catalytic site, to shield the donor substrate from hydrolysis and create an acceptor binding site [67] (Figure 3). The N-terminal domain of CstII ST possesses some sequence similarity with sialylmotif L of the eukaryotic ST [27]. This prediction was later reinforced by the elucidation of the 3D structure of the first mammalian ST, i.e., porcineST3GAL1 [33] (also named *Ss*ST3GalI), whose catalytic domain displays a mixed α/β fold with a seven-stranded parallel β-sheet flanked by 12 α-helices. PorcineST3GAL1 exhibits a modest 10% sequence identity with CstII but contains a β-sheet core and a lid-like structure analogous to the CstII fold. Porcine ST3GAL1 is speculated to be a second distinct GT-A variant (i.e., variant 2) (Figure 3), which displays a disulfide bond linking two conserved Cys residues of sialylmotifs L and S and mirrors the signature structure of the eukaryotic ST family [36,37,40]. Crystal structures of human STs have revealed that *Hs*ST3GAL1 [70], *Hs*ST6GAL1 [71], *Hs*ST6GALNAC2 [14], and *Hs*ST8SIA3 [72] adopt a GT-A variant 2 topology (Figure 3) and broadly resemble the fold ofporcineST3GAL1 [36,37,40] and ratST6GAL1 [73], i.e., a seven-stranded β-sheet flanked by multiple helices. Intriguingly, STs of the GT29 family have a histidine residue as acatalytic base, e.g., His-319 in porcineST3GAL1 [33], His-367 in ratST6GAL1 [73], His-316 in *Hs*ST3GAL1 [70], His-370 in *Hs*ST6GAL1 [71], His-351 in *Hs*ST6GALNAC2 [14], His-346 in *Hs*ST8SIA2, His-354 in *Hs*ST8SIA3, and His-331 in *Hs*ST8SIA4 [72] (Figure 4).

### 4.2. FUTs Display the GT-B Fold and Variations of It

Based on crystallographic data available for FUTs in mammals, FUTs appear to adopt variations of the GT-B fold. However, there are examples of FUTs that utilize residues from both domains to interact with acceptor substrates [59,60,61]. Strikingly, the geometry of the cleft has also been found to be modulated in order to accommodate extended branched-glycan structures.

**Figure 5:**
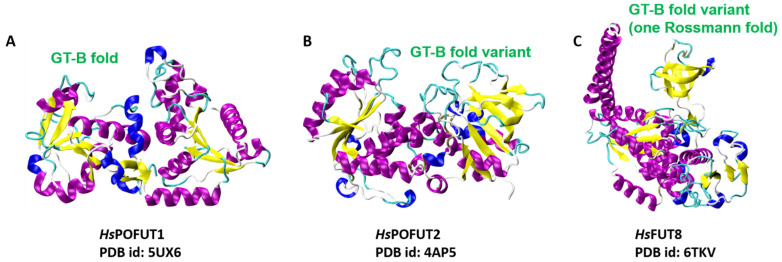
Representative glycosyltransferase with GT-B global fold and its variants. (**A**) GT-B fold corresponding to *Hs*POFUT1 (PDB id: 5UX6). (**B**) GT-B fold variant corresponding to *Hs*POFUT2 (PDB id: 4AP5). (**C**) GT-B fold variant with a single Rossmann fold corresponding to *Hs*FUTV8 (PDB id: 6TKV).

Crystal structures of FUTs in *Caenorhabditis elegans* and *Homo sapiens* illustrated that POFUT1 and POFUT2 display the GT-B fold and variations of it [61,74] (see Figure 5). In *Ce*POFUT1, the residues from the active site that interact with GDP-Fuc are mainly in the C-terminal domain together with those from the N-terminal domain [74]. The overall structure of *Hs*POFUT1 closely resembles that of *Ce*POFUT1; however, *Hs*POFUT2 has a variant of the GT-B fold in which the N- and C-terminal domains interact closely with each other to form an extended protein unit [59,60,61]. Li et al. reported the crystal structure of the mouse POFUT1 in a complex with both donor and acceptor substrates, i.e., GDP/GDP-Fuc and EGF-like domains (LDs), respectively [60]. EGF-LDs lie in the wide groove between the N- and C-terminal domains of the canonical GT-B fold in the ternary complex of *Mm*POFUT1:GDP:EGF-LD. Similarly, in another ternary complex between *Ce*POFUT2, GDP, and *Hs*TSR1 (the first TSR identified as human thrombospondin 1), GDP is found in a shallow cavity of the C-terminal domain. In contrast, half of *Hs*TSR1 is embraced by a cleft formed between both domains [61]. Intriguingly, the apo and complex structures of *Hs*FUT8 revealed that the GT-B fold contains only one Rossman fold (Figure 5), which contains a series of loops and an α-helix that contribute toward forming the ligand binding region [75].

## 5. Binding Interactions of STs with Natural Donor Substrate CMP-Neu5Ac

Meng et al. [73] crystallized the structure of ratST6GAL1 and described several conserved features shared by ratST6GAL1 with *Cj*CstII [76], porcineST3GAL1 [36,37,40], and *Hs*ST6GAL1, including the sialylmotif region involved in binding the donor substrate, i.e., CMP-Neu5Ac. Despite the fact that they were predicted to be similar to the CStII fold, the binding regions of mammalian ST structures display considerable variability with minimal conservation in the residues that directly interact with CMP-Neu5Ac regarding the *C. jejuni* CstII structure. *Cj*ST contains anNH2-terminal end that starts at the equivalent of sialylmotif L with an extended COOH-terminal sequence beyond the final β-strand, contributing to the catalytic domain and membrane tethering [76]. In contrast, the COOH-termini of mammalian STs terminate almost immediately after the final β-strand but display extended sequences on the NH2-terminal side of the sialylmotif, which contribute to both the catalytic domain and membrane tethering [36,37,40]. However, the sialylmotif sequences, which comprise the underlying scaffold of the Rossmann fold and adjoining loop regions, have been found to be conserved and are engaged in stabilizing the residues of the donor binding region within the *Cj* and mammalian STs [73].

**Figure 6:**
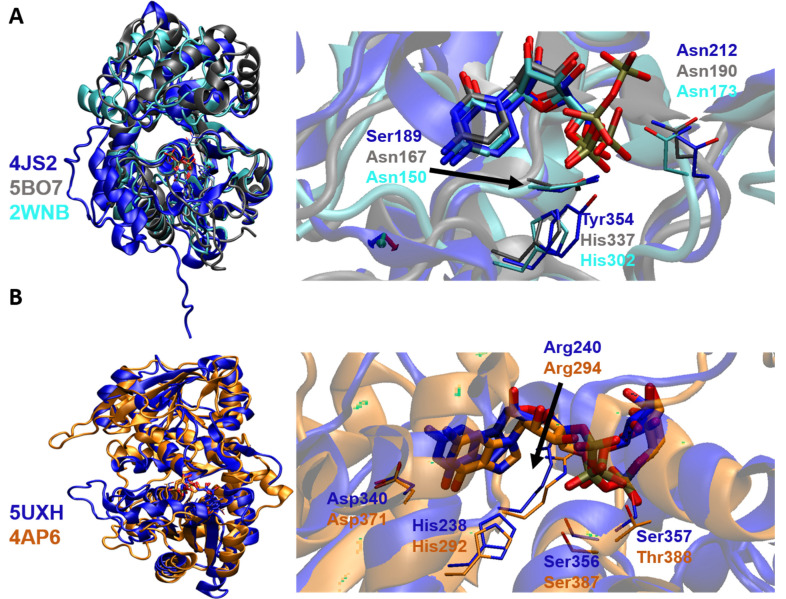
(**A**) Structural alignment of the protein backbone of three different sialyltransferases: *Hs*ST3GAL1 (cyan, PDB id: 2WNB), *Hs*ST6GAL1 (blue, PDB id: 4JS2), and *Hs*ST8SIA3 (gray, PDB id: 5BO6), highlighting the alignment of the donor CMP/CDP substrates and their surrounding residues shown in sticks. Zoomed views of the bound ligands with their interacting residues are displayed in the right panel. (**B**) Structural alignment of the protein backbone of the two FUT complexes. The ternary complex of *Hs*POFUT1 is shown in blue (PDB id: 5UXH) and *Hs*POFUT2 is shown in brown (PDB id: 4AP6), with the aligned GDP-fucose donor substrates and surrounding residues shown in sticks. Zoomed views of bound ligands with their interacting residues are displayed in the right panel.

It has been observed that CMP and CMP-Neu5Ac form multiple noncovalent interactions with the active site residues comprising the GT-A-defining nucleotide-binding Rossmann fold in *Hs*ST6GALNAC2 and *Hs*ST8SIA3, respectively. These interactions are similar to those observed for donor binding in bacterial CstII and other human STs, affirming that the sialylmotif scaffold underlying the CMP-NeuAc binding site is conserved [72,76]. Structural superimposition of *Hs*ST3GAL1, *Hs*ST6GAL1, and *Hs*ST8SIA3 clearly indicates that the donor substrate displays a similar orientation within the binding cleft and that the residues interacting with CMP or CDP are variable, while highly conserved amino acid residues are involved in recognizing the CMP or CDP of the donor substrate (Figure 6A).

Recently, Harrus, et al. solved the crystal structure of *Hs*ST6GAL1 in the apo- and CMP-Neu5Ac-bound states and reported on the flexibility of the catalytic loop [32]. The apo structure contains Tyr354, which interacts with the CMP-Neu5Ac at both the phosphate and Neu5Ac moieties, implying that the unliganded enzyme has an inherent interaction with the donor substrate. However, the bound state displays an alternate conformation, which prepares *Hs*ST6GAL1 to perform the hydrolysis step. Comparison of this new liganded structure [32] with the previously reported *Hs*ST6GAL1 [71] revealed the following differences: (a) the region 366–372, corresponding to motif “d” and sialylmotif VS, is unstable; however, binding to either (i) the acceptor substrate or (ii) α-helix 100–121, irrespective of the acceptor interaction, is speculated to stabilize this region; (b) the disulfide bond C353–C364 exists in a different orientation in the new structure, implying a movement in this region upon binding the acceptor; and (c) binding of CMP-Neu5Ac involves the side chain at C-5 of the sugar residue, which is directed toward empty space at the surface of *Hs*ST6GAL1. Interestingly, the exact binding mode of Neu5Ac directly involves thesialylmotifs L, S, and III, and transfers the sialylmotif VS into the immediate vicinity. Hydrophobic interactions, π-alkyl and amide π-stacking, and a multitude of hydrogen bonds stabilize the overall structure of *Hs*ST6GAL1 [32].

## 6. Binding Interactions between FUT and Its Natural Donor Substrate GDP-Fucose

Despite significant differences in sequence and domain architecture, the interaction of FUT with its donor substrate, GDP-Fuc, is analogous among the different human FUTs with solved structures to date. Interestingly, the residues interacting with the fucose moiety are variable, while highly conserved residues are involved in recognizing the nucleotide moiety of the donor substrate. For instance, in both *Hs*POFUT1 and *Hs*POFUT2, most residues interacting with the GDP of the donor fucose are conserved [74]. β-phosphate of GDP-Fuc interacts with Arg240 and Arg294 through hydrogen bonding and electrostatic interactions in both *Hs*POFUT1 and *Hs*POFUT2. Furthermore, the residues Asn46/Asn57, His238/His292, Asp340/Asp371, Ser356/Ser387, Ser357/Thr388, and Phe358/Phe389 interact with GDP in *Hs*POFUT1/*Hs*POFUT2, contributing to the tethering of the donor substrate (Figure 6B). However, the residues responsible for recognizing and stabilizing the fucose moiety, namely Arg43/Asp244 of *Hs*POFUT1 and Pro53/Gly55 of *Hs*POFUT2, are variable [74].

**Figure 7:**
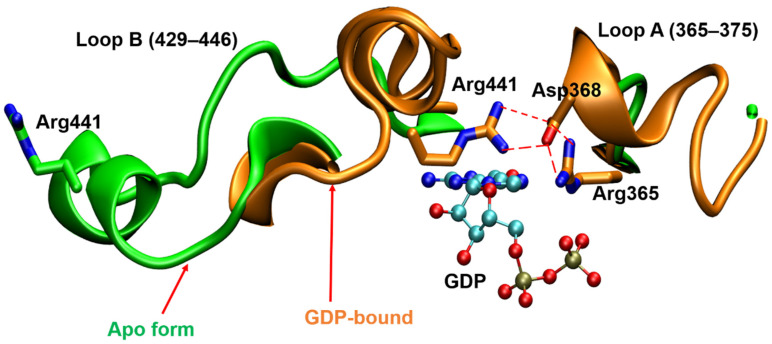
Superimposition of the GDP binding sites of apo form of *Hs*FUT8 (green, PDB ID:6VLE) and GDP-bound *Mm*FUT8 (brown, PDB ID:6VLG). The salt bridge interactions appear between Asp368 and Arg365/Arg441 from loops A and B, respectively, upon binding of GDP.

The binding interaction of *Hs*FUT8 with GDP-fucose was studied using computational techniques based on the binary complex of *Ce*POFUT1-GDP-fucose [77]. Since the binding sites of the donor molecule in *Ce*POFUT1 and *Hs*FUT8 are structurally similar, the investigators placed the donor molecule into the *Hs*FUT8 using the same positioning as seen in the structural relative *Ce*POFUT1. Analogous to *Hs*POFUT1 and *Hs*POFUT2, Arg365 interacts with the β-phosphate of GDP. Strikingly, Arg365 interacts with the fucose moiety in *Hs*FUT8, which has not been observed in human POFUTs. Further, Arg365 is speculated to assist the release of GDP and confer proper orientation of the fucose residue for the nucleophilic attack of the acceptor [77]. Jarva et al. solved the ternary complex of GDP:*Hs*FUT8:GlcNAc2Man3GlcNAc2-Asn(A2-Asn) and revealed another unique property of FUT8 in mammals, which is that it undergoes a conformational change upon binding to GDP [51]. Loop A (Arg365–Ala375) and loop B (Asp429–Asn446) are disordered in the unliganded *Hs*FUT8 structure but become ordered upon binding GDP. This transformation leads to the formation of new interactions between loops A and B; in particular, the electrostatic interactions between Asp368 and Arg365 of loop A and Arg441 of loop B are involved (see Figure 7). Arg365 forms a salt bridge with the β-phosphate of GDP. These findings imply that the binding of the GDP moiety with FUT8 reorganizes the encapsulating loops around the nucleotide. Additionally, the interactions involving the Asp453/His363-guanine base and the Tyr250-ribose hydroxyl groups are reported to contribute toward reorganizing both loops [51]. Later, Boruah et al. reinforced these findings that the loop regions are extended away from the donor binding site in the absence of GDP; however, the loops are flipped in to enclose the donor analog in the GDP:FUT8 complex [78]. It is notable that despite displaying this novel feature once the substrate is bound to FUTVIII, the spatial orientation and interactions with FUTVIII are nearly identical for other FUTs reported in mammals. These findings suggest that a common scaffold seems a promising approach to target human α1,6 FUT and O-FUTs [62].

## 7. Binding Interactions of STs and FUTs with Their Acceptor Substrates

Based on the nature of the acceptor substrate (i.e., glycan or protein), STs/ FUTs can be classified as glycan- or protein-modifying GTs. Glycan-modifying GTs include all ST and FUT subfamilies except the O-FUTs, which are protein-modifying GTs in humans.

### 7.1. Glycan-Modifying STs and FUTs

**Figure 8:**
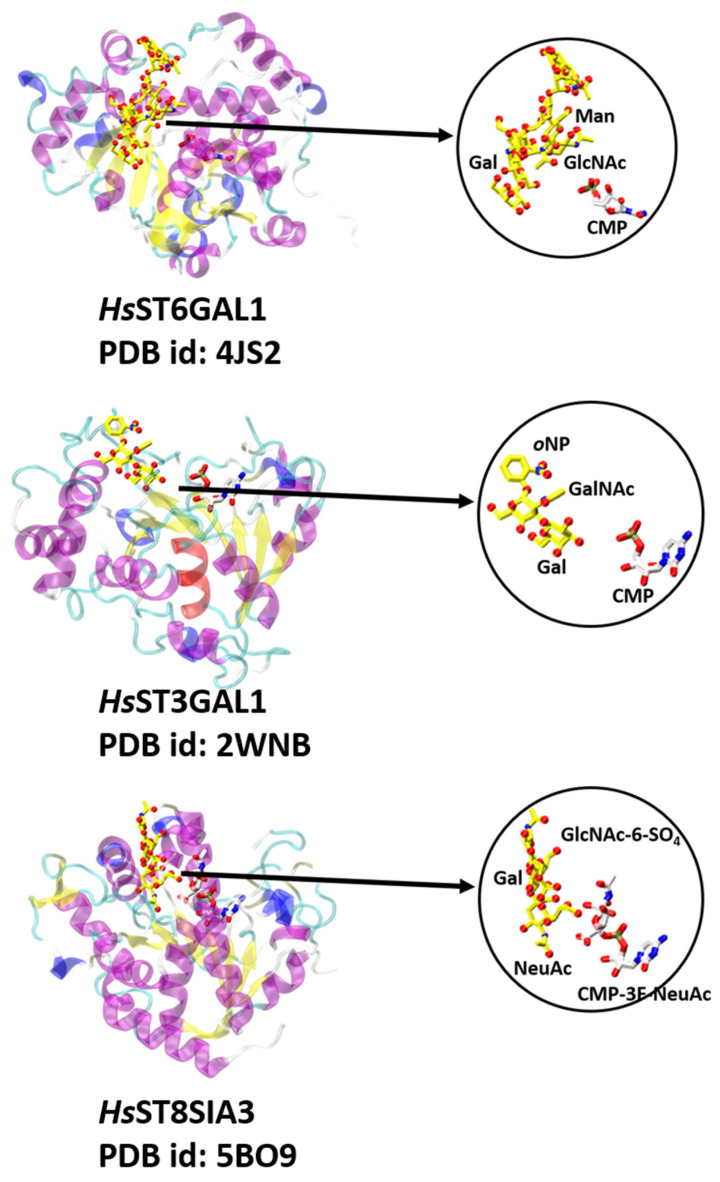
Three sialyltransferase structures with a GT-A fold (variant 2) shown in the circles with the bound donor analogs (white sticks) and acceptor analogs (yellow sticks). Structures include *Hs*ST6GAL1 (PDB id: 4JS2), *Hs*ST3GAL1 (PDB id: 2WNB), and *Hs*ST8SIA3 (PDB id: 5BO9). Close-up views of the bound donor and acceptor ligands are displayed in the same orientation and coloring as highlighted in the circled structures.

Glycan-modifying GTs include mammalian GT29 sialyltransferases that employ analogous conserved sugar donors but recognize diverse acceptor substrates. The acceptor binding regions of STs have shown striking differences in their sequence, secondary structure, and position among the members of the GT29 family [73]. The crystal structure of *Hs*ST6Gal1 bound to the Gal2GlcNAc2Man3GlcNAc2-Asn acceptor illustrates that the C6-OH group of the terminal Gal residue from the Gal-β-1,4-GlcNAc moiety of the acceptor is adjacent to the His catalytic base [72,73] (Figure 8). Interestingly, the *Hs*ST3Gal1-Gal-β-1,3-GalNAc-*o*NP binary complex revealed that the plane of the terminal Gal acceptor residue is rotated by 180°, which ultimately positions its C3-OH group adjacent to the catalytic His residue for glycan transfer [73] (Figure 8). This flipped geometry alters the nature of the interaction with the acceptor in *Hs*ST3Gal1. In fact, extensive hydrogen bonding stabilizes the complex while taking advantage of the down-facing axial hydroxyl groups of the disaccharide acceptor. These interactions are quite different from the mode of hydrophobic stacking and hydrogen bonding that is common among Gal-specific binding proteins, including *Hs*ST6GAL1 [73]. The ternary complex of CMP-3F-NeuAc-*Hs*ST8SIA3-NeuAc-α-2,3-Gal-β-1,4-GlcNAc-6-SO4 showed that the acceptor NeuAc of ST8SIA3 primarily forms hydrogen-bonding interactions by positioning the nucleophilic C8-OH group of NeuAc adjacent to the His catalytic base [72] (Figure 8). The crystal structure of the CMP-*Hs*ST6GALNAC2 binary complex, but without an acceptor, revealed minimal sequence and structural similarity in the primary sequence of the loop regions and secondary structural elements involved in acceptor substrate recognition compared to *Hs*ST6GAL1, *Hs*ST3GAL1, and *Hs*ST8SIA3, implying the presence of significant diversity in the acceptor binding region among members of the GT29 family [14].

**Figure 9:**
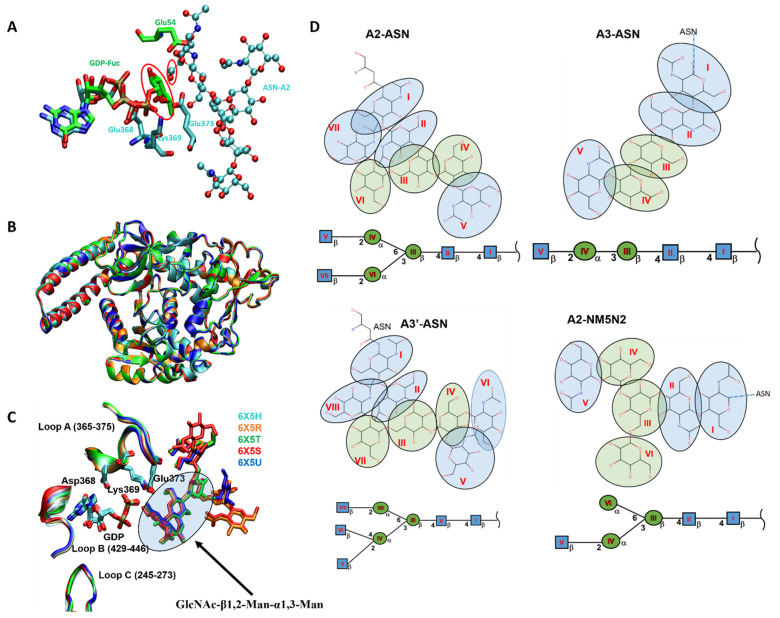
(**A**) The molecular interaction of *Hs*FUT8 with its substrates and its catalytic mechanism in humans. The GDP:*Hs*FUT8:A2-Asn ternary complex (PDB id:6X5R)is aligned with the GDP-Fuc:*Hs*POFUT2 binary complex (PDB id: 4AP6). The aligned structures overlay the nucleotide and ribose (cyan sticks for GDP bound to FUT8 and green sticks for GDP-Fuc bound to POFUT2) in both structures and place the fucose (green-circled sticks) in the active site of FUT8 for the fucose residue which is aligned for nucleophilic attack by the OH-6 of the GlcNAc-1 of A2-Asn. Both the fucose and OH-6 GlcNAc nucleophile are circled in red, and Glu373 is the catalytic base. (**B**) Alignment of human FUT8 in the presence of a donor substrate analog and with four distinct glycan acceptors. Cyan represents no glycan, orange represents ASN-A2 glycan, green represents ASN-A3, red represents ASN-A3′, and blue represents ASN-NM5N2 glycan. (**C**) Close-up image of the active site of FUT8 in the presence of a donor substrate analog and four distinct glycan acceptors (superimposed). (**D**) Schematic representation of glycan acceptor structures.

The crystallization of the GDP:FUT8:GlcNAc2Man3GlcNAc2-Asn ternary complex was a milestone toward understanding the interaction of FUT8 with its substrates and catalysis in humans. FUT8, a GT involved in the core fucosylation of mammalian branched N-glycans, has been extensively explored to identify conserved and divergent structural features for acceptor recognition employing the ternary complex. FUT8 features an N-terminal coiled-coil domain, a catalytic domain, and a C-terminal SH3 domain, a unique characteristic among GT proteins [62]. The ternary complex of GDP:*Hs*FUT8:GlcNAc2Man3GlcNAc2-Asn (A2-Asn),investigated by Kotzler et al., displays that a hexasaccharide is required as a minimal acceptor structure [79]. The 6-OH of the GlcNAc-1 of the hexasaccharide must be nearby the anomeric position of the Fuc residue of the bound donor for its transfer to the 6-OH of the GlcNAc-1 of the acceptor. The hydrogen bonds and hydrophobic interactions of the acceptor with GDP-Fuc contribute significantly to the binding of the acceptor. The branch at the 3-position mannose is bound through multiple hydrogen bonds between the flexible loop and the C-terminal β-sheets. Despite being distant from the site of the fucosyl transfer, GlcNAc-5 at the 3-mannose branch is essential for substrate specificity and exhibits transient interactions with the Lys541 side chain and with the flexible loop. The carboxyl group of Glu373 is essential for catalytic activity by engaging in interactions with the OH-3 and OH-4 of GlcNAc-5 [79]. Recently, novel insights have been made regarding *Hs*FUT8 glycan acceptor recognition. Gracia et al. captured a ternary complex of GDP:*Hs*FUT8:A2-Asn and showed that the catalytic domain is connected to the N-terminal coiled-coil domain by interdomain α3; however, the C-terminal SH3 domain is in contact with the catalytic domain by the β10–β11 loop [75]. GDP is partly buried and confined within the catalytic domain. GlcNAc-1 and -2 of the core region of A2-Asn are also present in the catalytic domain. However, α3/α6-branches of A2-Asn are located in the exosite formed by the β10–β11 loop and the SH3 domain. Interestingly, the β6–α8 loop (residues 365–378) is partly disordered in the apo form, but undergoes a conformational change in the presence of substrates; thus, the key residues Arg365, Lys369, and Glu373 not only recognize GDP and A2-Asn, but also contribute to catalysis since Glu373 acts as the catalytic base for the 6-OH group of the GlcNAc-1 of A2-Asn [75]. When the GDP:*Hs*FUT8:A2-Asn ternary complex is aligned with the GDP-Fuc:*Hs*POFUT2 binary complex, the fucose residue from donor substrates occupies the appropriate position within the active site of FUT8 such that C1 of the Fuc residue is directly aligned for nucleophilic attack by the OH-6 of the GlcNAc-1 of A2-Asn (Figure 9A), as recently demonstrated [62].

To further understand the preferences of the acceptor site, Jarva et al. solved the ternary structure of FUT8 in both mice and humans. The SH3 domain emerged as contributing toward the evolution of FUT8 as a dimer, which restricts the movement of the SH3 domain and stabilizes the acceptor binding site [51]. Glu373 displays close hydrogen bonding with the 6-OH group of the GlcNAc residue of A2-Asn, which interacts with Lys369 and in turn is in close contact with the β-phosphate of GDP. Notably, the activity of FUT8 depends on the terminal GlcNAc of the α3-branch since the intimate hydrogen bonding between His353 and the 6-OH group of this GlcNAc contributes toward acceptor binding. In another study, Boruah et al. performed kinetic studies on *Hs*FUT8 with the acceptor A2-Asn and its structural analogs to investigate the restricted substrate recognition of the enzyme [78]. The structural superimposition of *Hs*FUT8 bound to the donor substrate analog with four distinct glycans (i.e., A1-Asn, A2-Asn, A3′-Asn, and NM5N2-Asn) corroborates their observation that the trisaccharide GlcNAc-β1,2-Man-α1,3-Man moiety is the key determinant for the acceptor recognition of FUT8 [78] (Figure 9B,C). It is notable that FUT8 displays a highly rigid active site that allows access to only a few potential structures, despite having a common Man3GlcNAc2-Asn structure in the core region of these acceptor substrates.

### 7.2. Protein-Modifying FUTs

Protein-modifying GTs target the hydroxyl group of Ser or Thr in proteins. They must first bind to the acceptor protein to orient the respective Ser/Thr hydroxyl nucleophile correctly for the transfer of a sugar moiety from the donor substrate. These include POFUTs, which modify folded cysteine-rich domains [14]. POFUT1 transfers fucose to the Ser or Thr residue of EGF repeats containing the consensus sequence C^2^-X-X-X-X-(S/T)-C^3^ [80]. However, POFUT2 glycosylates Ser or Thr residues in the consensus sequence C^1^-X-X-(S/T)-C^2^ or C^2^-X-X-(S/T)-C^3^ of TSRs of groups 1 and 2, respectively (where X is any amino acid) [80]. While disulfide bridges of group 1 TSRs follow the pattern C^1^–C^5^, C^2^–C^6^, and C^3^–C^4^, the TSRs of group 2 are arranged as C^1^–C^4^, C^2^–C^5^, and C^3^–C^6^. Interestingly, the binding region of each of these O-FUTs is complementary to the face of the domain of the protein, which interacts with the cleft through multiple hydrogen bonds, especially within the loop with the consensus sequence. This orients the domain to transfer the OH-group of the Ser/Thr acceptor substrate exactly in the correct position to perform the nucleophilic attack on the anomeric C-1 of the Fuc residue of the donor substrate [60,61].

Experimental structures of *Hs*POFUTs in complexes with their acceptor substrates are not yet solved; however, the ternary complexes of O-FUTs have been crystallized in mice and *C. elegans*. The ternary complex of GDP/GDP-Fuc:*Mm*POFUT1:EGF-LDs revealed multiple points of interaction between POFUT1 and EGF-LDs. However, the core of the interaction involves a conserved preformed cleft on POFUT1 and conserved, sequence-independent structural elements on the fucosylation motif, which is common to all EGF-LDs [60]. The interactions outside the core region display POFUT1 residues, which are flexible, and EGF-LD residues that are highly variable among POFUT1 substrates. Thus, the observed plasticity of *Mm*POFUT1 is an important feature, which enables the enzyme to accommodate the sequence diversity of its EGF-LD substrates. The structure of *Ce*POFUT2–GDP–*Hs*TSR1 highlighted that the *Ce*POFUT2 binding domain contains large cavities that are filled by an intricate network of water molecules [61]. The complex is stabilized by a limited number of direct hydrogen bonds and stacking interactions between *Ce*POFUT2 and *Hs*TSR1 that are complemented by many water-mediated interactions. The proactive role of these water molecules is speculated to bestow promiscuity to *Ce*POFUT2 toward dissimilar TSRs, which might be claimed for similar GTs that modify a wide variety of peptide sequences.

## 8. Mechanism of Catalysis

**Figure 10:**
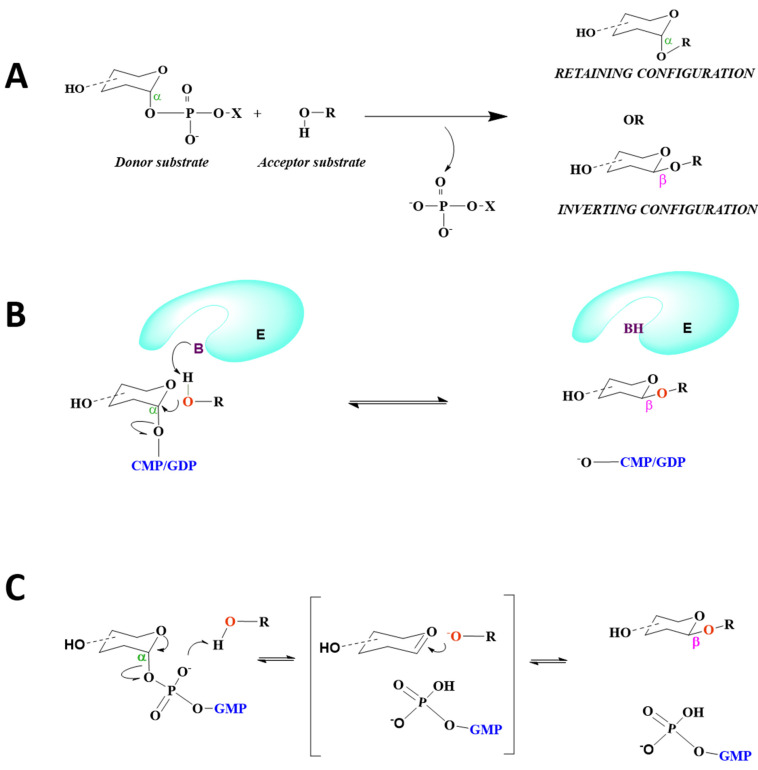
Reaction mechanism of STs and FUTs in mammals. (**A**) GTs catalyze the transfer of a sugar residue from a donor substrate to an acceptor substrate either by retaining or inverting the anomeric stereochemistry with respect to the donor sugar. (**B**) S_N_2 inverting mechanism of theGT29 family (e.g., *Hs*ST3GAL1, *Hs*ST6GAL1, *Hs*ST8SIA2, *Hs*ST8SIA3, *Hs*ST8SIA4, and FUTs) (e.g., *Ce*POFUT2, *Hs*POFUT2, and*Hs*FUT8). (**C**) S_N_1 inverting mechanism for *Ce*POFUT1 and *Mm*POFUT1. The cyan E displays the enzyme (ST/FUT); the purple B represents the catalytic base, which deprotonates the acceptor nucleophile; and the red O indicates the nucleophilic oxygen of the acceptor substrate. CMP, GMP, and GDP (blue) are cytidine monophosphate, guanosine monophosphate, and guanosine diphosphate, respectively.

The catalytic mechanism is independent of the overall fold of GTs since both inverting and retaining enzymes are common among the GT-A and GT-B superfamilies. Nucleotide-dependent GTs catalyze a glycosyl transfer reaction either by retention or inversion of stereochemistry at the anomeric reaction center of the donor substrate to generate diverse biological glycans with distinct anomeric configurations [8] (Figure 10A). The orientation of the acceptor hydroxyl group relative to the donor anomeric carbon is the critical step in establishing the catalytic mechanism for GTs.

### 8.1. STs Display S_N_2 Catalysis

STs are classified as metal-ion-independent inverting enzymes that employ an S_N_2 single-displacement reaction mechanism (Figure 10B), in which the nucleophilic hydroxyl group of the acceptor attacks the anomeric carbon of the donor sugar, i.e., sialic acid, and a catalytic base assists in the deprotonation of the nucleophile; the nucleotide moiety leaves from the opposite face, resulting in the inversion of the anomeric configuration of the product [16]. As aforementioned, histidines appear to serve as catalytic bases for the sialyltransfer reaction of GT29 STs, thus emphasizing the role of histidine as an important base in their catalytic mechanism (Figure 4).

### 8.2. FUTs Display S_N_1 or S_N_2 Catalysis

Analogous to the ST family, the S_N_2 single-displacement reaction mechanism is characteristic of fucosyltransferases such as *Ce*POFUT2^61^ and *Hs*POFUT2 [59] (Figure 10B). Despite the absence of ligands, FUTs utilizing an S_N_2 inverting mechanism usually contain catalytic residues located in their binding pocket, such as Glu52 in *Ce*POFUT2 [61] and Glu54 in *Hs*POFUT2 [59]. Contrary to POFUT2, an S_N_1 mechanism involving the formation of a close ion pair is postulated for POFUT1, in which the glycosidic bond is cleaved before the nucleophilic attack [81](Figure 10C). In *Ce*POFUT1, Asn43 may be positioned at the hydroxyl group of the acceptor, close to the β-phosphate. However, Arg240 is the key catalytic residue, through which hydrogen bonding with the glycosidic-bound oxygen may facilitate the cleavage of the glycosidic bond. Additionally, in *Mm*POFUT1, Asn51 and Arg245 are engaged in hydrogen bonding with acceptor molecules and the β- phosphate group, respectively [60]. It is notable that no basic residues are present in the active sites for *Ce*POFUT1 and *Mm*POFUT1 that could act as assisting bases.

Based on in silico modeling of GDP-Fuc-bound *Hs*FUT8 with A2Asn, an S_N_2-like reaction is predicted for the enzyme, in which β-phosphate is speculated to play the role of the catalytic base [77,79]. Subsequently, the GDP:*Hs*FUT8:A2-Asn crystal structure revealed that the motion of the β6–α8 loop brings the essential catalytic base Glu373 to the binding site in the presence of ligands [75], information which was not evident from previous computational studies [77,79], thus emphasizing the need to directly capture donor-enzyme-acceptor ternary complexes to delineate the molecular basis of catalysis for GTs.

## 9. Molecular Interactions of Porcine ST3GAL1 with a Potent Inhibitor That Mitigates Tumor Cell Metastasis

**Figure 11:**
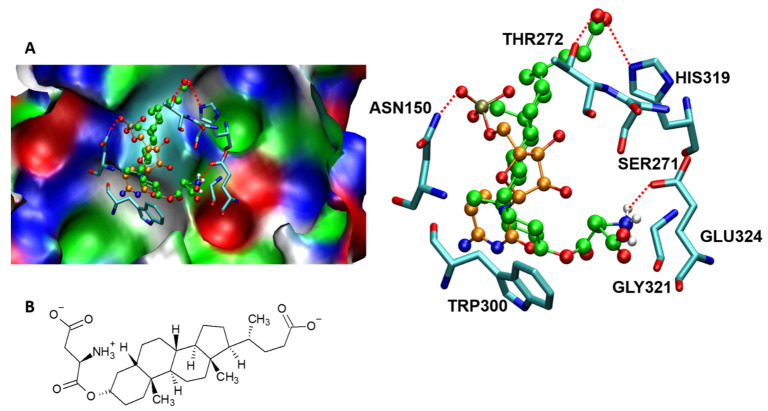
(**A**) The molecular docking of Lith-O-Asp to mammalian sialyltransferase (PDB ID: 2WNB). Lith-O-Asp is shown in green and CMP is shown in orange. Hydrogen bonds are shown with dotted red lines. The highlighted interactions between Lith-O-Asp and amino acids of the binding sites are shown in the right panel. (**B**) Structure of Lith-O-Asp used as an inhibitor for the docking study.

Cells treated with a lithocholic acid analog (Lith-O-Asp) revealed reduced activities of ST3GAL1, ST3GAL3, and ST6GAL1 in in vitro and cell-based activity analyses [81]. Lith-O-Asp has been proposed to abrogate tumor cell metastasis, partly by inhibiting ST activity to attenuate the expression of cell-surface sialylated antigens such as integrin-β1 and inhibit FAK/paxillin/Rho signaling activity in vivo [81]. To understand the nature of the interaction between the mammalian ST and Lith-O-Asp, we performed a molecular docking analysis between porcineST3GAL1 (PDB ID: 2WNB) and Lith-O-Asp that clearly revealed the strong binding affinity of Lith-O-Asp with active site amino acid residues with an interaction energy of −8.75 kcal/mol (Figure 11). Our computational analysis revealed major interactions stabilizing the enzyme-inhibitor complex, which include: (a) a close interaction of the acidic end of Lith-O-Asp and His319, Thr 272, and Tyr233 of ST3GALI, and (b) interaction of the amine end of Lith-O-Asp with Glu324. Recently, Ortiz-Soto et al. [70] generated a model of *Hs*ST3GAL1 based on the ternary porcine ST3GAL1 complex and investigated the molecular interactions between the enzyme and its substrates to further understand the correlations among the structure, activity, and stability of ST3GAL1 in humans. The removal of hydrogen bonds and/or stacking interactions among both donor and acceptor substrates and residues such as Tyr191, Tyr230, Asn147, Ser148, and Asn170 influences the activity of ST3GAL1 to different extents. Intriguingly, the removal of disulphide Cys59–Cys64 reduces the activity of donor and acceptor substrates in vitro. Here, computational techniques could be employed to gain insight into the interactions of ST3GAL1 with its substrates to provide a theoretical model to further evaluate the interaction of Lith-O-Asp and similar metabolic inhibitors with ST3GAL1 and other sialyltransferases in humans. Thus, a computational biology approach toward developing selective therapeutic targets may act as a catalyst for drug discovery in cancer.

## 10. Structural Modeling of STs and FUTs

Molecular modeling of STs and FUTs is challenging since the number of available crystal structures remains limited for humans. Previously, in the absence of crystal structures, homology modeling along with fold recognition or threading techniques were used to predict structures. The first structural model for the mammalian FUT family, FUT4 in mice, was developed using this technique [82]. Furthermore, homology models have been proposed for *Hs*FUT3 and *Hs*FUT7, as described in detail by de Vries et al. [41]. Homology models for *Hs*ST8SIA1 and *Hs*ST8SIA4 have also been developed with RosettaCM50 using a template alignment generated with Modeller [72]. Additionally, the interaction of *Hs*ST8SIA4 with its acceptor substrate has been explored with RosettaDock [72]. The SWISS-MODEL server has been used to generate a homology model of *Hs*ST3GAL1using the crystal structure of porcine ST3GAL [70]. Strecker et al. used Schrodinger’s Maestro software to recreate a model of *Hs*FUT8 for donor substrate binding using its crystal structure [83]. In addition, a molecular dynamic (MD) simulation has been performed with Desmond v3 to explore the flexibility of *Hs*FUT8 [83]. Since both STs and FUTs are inverting glycosyltransferases that involve large conformational movements, we propose that the MD simulation techniques could be useful to study the flexible loops that are proposed in their catalysis.

## 11. Conclusions

STs and FUTs display conformational plasticity upon substrate binding and catalysis. Notable progress has been made in the last decade in the successful crystallization and molecular modeling of several STs and FUTs, which has opened new avenues to fill gaps in our understanding of their structural architecture, interactions with substrates, and catalytic mechanisms. This progress holds the promise to impact medical and biotechnological development. Despite the remarkable breakthrough in the determination of high-resolution crystal structures of mammalian STs and FUTs in both apo and binary complexes, very few are available as ternary complexes. It is therefore essential to capture donor-enzyme-acceptor ternary complexes to delineate the molecular basis of the catalytic mechanisms of these glycosyltransferases, which are sought-after therapeutic targets in diseases such as cancer.

## Figures and Tables

**Table 1:** Features of known STs and FUTs in humans and upregulated expression in cancers.

Enzyme Family	Enzyme	CAZy Family	Global Fold	Donor Substrate	Preferred Acceptor Substrate	Upregulated Expression in Cell Lines or Tissues
ST3GAL	ST3GAL1	GT29	GT-A	CMP-Neu5Ac	Galβ1, 3GalNAc	Melanoma, breast, ovarian, and liver cancers
	ST3GAL2				Galβ1, 3GalNAc	-
	ST3GAL3				Galβ1, 3(4)GlcNAc	Melanoma, glioma, breast, and pancreatic cancers
	ST3GAL4				Galβ1, 4(3)GlcNAc	Gastric cancer
	ST3GAL5				Galβ1, 4Glc-ceramide	-
	ST3GAL6				Galβ1, 4GlcNAc	Multiple myeloma and gastric cancer
ST6GAL	ST6GAL1				Galβ1, 4GlcNAc	Liver, gastric, colon, bone, pancreatic, lung, prostate, breast, and ovarian cancers
	ST6GAL2				Galβ1, 4GlcNAc	Breast cancer
ST6GALNAC	STGALNAC1				GalβNAc-Ser/Thr and Galβ1,3GalNAc- Ser/Thr	Liver and gastric cancers
	STGALNAC2				Galβ1, 3GalNAc-Ser/Thr	Thyroid and colorectal cancers
	STGALNAC3				Siaα2, 3Galβ1, 3GalNAc-ceramide Siaα2, 3Galβ1, 3GalNAc-Ser/Thr	-
	STGALNAC4				Siaα2, 3Galβ1, 3GalNAc-Ser/Thr	-
	STGALNAC5				GM1b	-
	STGALNAC6				All α-series gangliosides	-
ST8SIA	ST8SIA1				Siaα2,3Galβ1,4Glc-ceramide	Breast cancer
	ST8SIA2				(Siaα2,8)nSiaα2,3Gal	-
	ST8SIA3				Siaα2,3Galβ1,4GlcNAc	-
	ST8SIA4				(Siaα2,8)nSiaα2, 3Gal	Breast cancer, acute myeloid leukemia
	ST8SIA5				GM1b, GT1b, GD1a, GD3	-
	ST8SIA6				Siaα2, 3Gal	Breast, lung, and liver cancers
α1,2 FUT	FUT1	GT11	GT-B	GDP-Fuc	Galβ1, 4GlcNAc	Bladder, breast, epidermoid, ovarian, and prostate cancers
	FUT2				Galβ1, 3GlcNAc	-
α1,3/4 FUT	FUT3	GT10			(Both sialyl- and non-sialyl-) Galβ1,3GlcNAc and Galβ1,4GlcNAc	Pancreatic, gastric, colorectal, oral, and head and neck cancers
	FUT4				Galβ1, 4GlcNAc	Melanoma, breast, and lung cancers
	FUT5				(Both sialyl- and non-sialyl-) Galβ1,3GlcNAc and Galβ1,4GlcNAc	Colorectal and gastric cancers
	FUT6				(Both sialyl- and non-sialyl-) Galβ1, 4GlcNAc	Liver, colorectal, prostate, oral, andhead and neck cancers; prostate cancer metastasis to bone
	FUT7				(sialyl-) Galβ1, 4GlcNAc	Liver, prostate, and lung cancers
	FUT9				Galβ1, 4GlcNAc	-
	FUT10				GlcNAc β1, 4GlcNAc-Asn	-
	FUT11				GlcNAc β1, 4GlcNAc-Asn	-
α1,6 FUT	FUT8	GT23			GlcNAcβ1, 2Manα1, 6[GlcNAcβ1,2Manα1,3]Manβ1,4GlcNAcβ1,4GlcNAc-Asn	Melanoma, lung, liver, breast, prostate, ovarian, cervical, and colorectal cancers
O-FUT	POFUT1	GT65			EGF-like repeats	Liver cancer
O-FUT	POFUT2	GT68			Thrombospondin type 1 repeats	
